# Implementing a patient-initiated review system in rheumatoid arthritis: a qualitative evaluation

**DOI:** 10.1186/s12913-015-0837-9

**Published:** 2015-04-15

**Authors:** Sue Child, Victoria A Goodwin, Mark G Perry, Christian A Gericke, Richard Byng

**Affiliations:** PenCLAHRC, Plymouth University Schools of Medicine and Dentistry, ITTC Building, Tamar Science Park, Drake Circus, Plymouth, Devon PL4 8AA UK; PenCLAHRC, University of Exeter Medical School, University of Exeter, Veysey Building, Salmon Pool Lane, Exeter, EX2 4SG UK; Department of Rheumatology, Plymouth Hospitals NHS Trust, Derriford Road, Plymouth, Devon PL6 8DH UK; The Wesley Research Institute, University of Queensland Schools of Medicine and Population Health and Queensland Brain Institute, PO Box 499, Brisbane, QLD 4066 Australia

**Keywords:** Rheumatoid arthritis, Patient-initiated, Follow-up, Direct access

## Abstract

**Background:**

The management of Rheumatoid Arthritis (RA), a chronic relapsing condition primarily affecting joints usually entails regular hospital reviews with a specialist. These reviews can occur when the patient is well. This study forms part of a service evaluation of a system wide implementation of a patient initiated review appointment system which we have called Direct Access (DA). The aim was to explore the experiences of patients and staff of a DA system in order to understand the process of the implementation and to identify potential improvements.

**Methods:**

Twenty-three patients with RA that had completed one year of follow-up on the DA system and seven healthcare professionals (HCPs) involved in the implementation of the DA review system took part in semi-structured interviews. Thematic analysis was used to analyse the interview data and field notes.

**Results:**

Four themes emerged in the data: (1) building patient confidence and empowerment, (2) right place right time, (3) safety, (4) the everyday challenges of managing change. These show that in order for implementation to be successful the patient needs to have confidence in using a new system of requesting a medical review, which, in turn, needs to be offered quickly and in a setting convenient to both patient and clinician. Embedded in the change process need to be systems for ensuring regular disease monitoring and general issues surrounding team working, communication and ownership of the change process also need to be considered from the outset.

**Conclusion:**

The clinics offer increased patient autonomy and the opportunity for greater self-management of chronic disease. This fits with new models of care where the patient is considered to be ‘the expert’.

## Background

Rheumatoid arthritis (RA) is a long-term condition that primarily affects the synovial joints causing unpredictable episodes of joint pain, swelling and stiffness. This results in disability and reduced quality of life. The condition affects around 400,000 people in the UK and is more common in women than men by a factor of 3:1 [1].

The system of follow up is traditionally service-driven by regular hospital reviews initiated by the rheumatologist or nurse specialist, or the availability of appointments in a schedule. Often, these pre-booked appointments take place when the patient is well and little, if any, action is needed [2]. The current system is not responsive to fluctuating disease and distress thus contributing to a mismatch between patient need and clinical input [2].

An alternative model of follow-up whereby patients initiate appointments themselves has been evaluated in a randomised controlled trial over six years [3-5]. The study found that the patient-initiated reviews were more efficient in terms of resource use, without detriment to patients’ physical and psychological status. These findings were used as the basis for implementing Direct Access (DA) clinics for adults that had had RA for at least two years, in one hospital in South West England which has been evaluated using a waiting list controlled, randomised controlled trial [6]. The proposed DA service comprised three main components:

- A small group education session led by a rheumatology nurse specialist to explain new access appointment arrangements;
- Access to a telephone advice line where patients could leave a message and would be contacted by the nurse specialist within one to two working days;
- Where indicated, access to a specialist appointment, with the rheumatologist or nurse specialist, within ten working days.

Those allocated to follow-up through the DA appointment system did not receive further regular appointments once they had attended the education session. All patients who had not requested or attended an appointment after twelve months were contacted for a clinical review. Those allocated to the waiting list control continued to receive regular, clinician (RC) driven appointments as in the previous traditional system. After twelve months, controls transferred to DA.

This study aimed to establish the experiences of the DA model of service delivery for patients with RA from the perspective of patients and those involved in service delivery in order to support interpretation of the wider study findings and identify further means of improving implementation.

## Methods

The National Research Ethics Service South-West (Bristol), UK advised that the study did not require NHS ethical approvals as it was a service evaluation. However, all interview respondents (both patients and professionals) were volunteers and informed consent was gained using standardised consent forms. All respondents were provided with a detailed information sheet which outlined the nature of the study and were given the opportunity to ask questions prior to interview. All interview transcripts were anonymised and confidentiality of the respondents was assured.

### Patients

All patients with RA that had completed one year of follow-up on the DA system were invited, in writing by the clinical team, to take part in a semi-structured interview. This timescale was chosen in order to allow a reasonable timeframe of experience of the new system. The invitation included a participant information sheet and a consent form. Those that were interested returned the signed informed consent form in a stamped addressed envelope to the researcher who then made contact to arrange a convenient time and preference for interview (telephone or face-to-face). Each respondent, (both telephone or face-to-face) was offered a £10 voucher as compensation for their time at the end of the interview.

### Staff

Staff from the rheumatology service were invited to take part in a semi-structured interview. The clinician that initiated the service development was interviewed and snowballing was then used to identify other key individuals involved in service delivery. These interviews were undertaken, following written informed consent, during the course of the working day at a time and place within the hospital setting convenient to individual working arrangements. Staff did not receive any financial incentive to take part in the interview.

All of the interviews focussed on experiences of both the DA and RC approaches. In particular, we were keen to understand how participants perceived the different service models and what impact it may have had on them or others.

An interview guide was used, and all respondents were given the opportunity to talk freely about their experiences at any time during the interview. All interviews were audio-recorded, transcribed verbatim and checked for accuracy. A provisional sample size of up to twenty five participants was proposed in order to achieve data saturation [7].

In order to give the researcher (who had no clinical background or experience of working with people with RA) an understanding of the context of DA, observation of the education sessions was undertaken [8]. These observed one hour sessions which were run by an experienced rheumatology nurse and delivered to small groups of patients prior to their follow-up appointments shifting from RC to DA clinics. Six sessions were observed and field notes made. By attending these sessions we obtained a better understanding of the process through which the patient learned about the new DA follow-up system.

Thematic analysis was used to analyse the data [9]. This involved two researchers (SC and VG) independently coding the interview transcripts and then identifying themes that were refined in collaboration with a third researcher (RB). Field notes from the education sessions were incorporated where deemed relevant. Thematic analysis is often used in health research [10]. It is an effective way of identifying key elements of experience from a personal account from a respondent. It involves identifying and analysing commonalities within data [11].

## Results

In total 23 patients agreed to take part (6 male, 17 female). Mean age (standard deviation) was 65.6 years (9.1) with a range of 50 to 84 years. Disease duration was a mean (SD) of 19.6 years (12.2) and a range of 4 to 40 years. All but one requested a telephone interview. Seven healthcare professionals agreed to take part. These included four rheumatologists (3 male, 1 female), two rheumatology nurse specialists (both female) and one administrator (female). One member of staff who was heavily involved in the implementation of DA completed two interviews fifteen months apart to give a perspective on how things may have changed during the implementation period. Fifteen patients recalled having called the telephone helpline. The mean (SD) number of calls per respondent over twelve months was 1.3 (1.5) with a range of 0 to 6 calls, whilst seven respondents indicated they had not needed to use the system at all.

Four main themes were identified. These were: (1) building patient confidence and empowerment, (2) right place - right time, (3) safety and (4) everyday challenges of managing change.

### Building patient confidence and empowerment

Attendance at an education session led by an experienced rheumatology nurse was the primary means for ensuring patients fully understood how the DA review system worked in practice. The number of patients attending each session varied between six and eight. During the session it was explained how the DA review system would operate and how appointments would be now scheduled by patients according to their needs, in addition to outlining how the telephone advice line would work. The attendees were then given the opportunity to ask questions about the delivery of a DA review system in practice.

Observations of the education sessions showed that initially patients appeared to be very sceptical of the DA system of follow-up with some people voicing their concerns that the change to the DA system of follow-up appeared to be a mechanism through which to discharge them from hospital waiting lists and shift provision of care back to GP. However, patients appeared to feel more reassured as the sessions progressed. These concerns were consistently voiced in all observed sessions and it appeared critical that the nurse delivering the education was well known to and trusted by all attendees. These observations were supported in the interviews:

“… I trust [nurse], I’ve known her for a long, long time and I trust her implicitly.” (R10).

“…I was very reluctant [to join DA]… I was a bit nervous really thinking Oh if I’m not going to get to see someone regularly…but then we went to the hospital [education session] and I felt much more reassured” (R14).

Patients on the whole also understood the rationale for the DA system:

“…so by being a direct access patient if I need anything when it goes wrong I know I can get help immediately which is at least a lot off my mind.” (R10).

Even though patients thought the education sessions were a very helpful way of explaining the shift from RC to DA review, some were still confused about how the advice line would operate and were still waiting for written instructions.

“… she promised to send out sort of explanatory notes about the whole system of direct access but I don’t know, I never got it, I’m still a bit mystified … I don’t really know what the difference is…” (R22).

The majority of patients welcomed the opportunity to self-manage their illness and reduce dependence on HCPs:

“…I do feel empowered by direct access because you have control of saying ‘ well actually I am not very well I need to see someone’ or ‘well, yeah, it’s a flare up and it will go down and yeah I can manage that and it’s so much better’. It is empowering.” (R10).

For some, the loss of regular monitoring at clinician-driven appointments was a concern whilst another patient appeared worried about continuity of care under DA. Her concerns surrounded the fact that deterioration might go unnoticed whereas a regular clinic would offer the opportunity to pick this up more quickly.

“I think everybody should have at least six monthly reviews…” (R6)

So whilst most patients were convinced during the sessions, especially by the presence of a trusted nurse, an underlying concern about being without support remained for a minority.

### Right place - right time

Being able to access care when needed was the most important objective of DA. Under the traditional system of follow up substantial amounts of time appeared to be wasted. For example, patients were often called for a review when they were well, did not need medical attention and were kept waiting for over-running clinics.

“…I’ve got to catch 2 buses which takes about an hour and a half to see a consultant for about 5 minutes, which is my estimation is a complete waste of time unless there is something wrong with me” (R9).

Both patients and staff considered that more timely access to care was a positive impact of DA:

“…if you are in pain and you want to see a rheumatologist you’ve got to get an appointment and that could take two or three months but this system here within 10 days you get to see a rheumatologist and obviously that’s a lot better than having to wait 3 months if you are in pain” (R2).

All patients and staff indicated in the interviews that they supported a change to DA for this reason:

“Well basically I think when you’ve had a condition like this for so long I mean you tend to know your body better than anybody else and I mean sometimes you are going for check-ups and you know well the thing is you don’t really need a check-up you know you’re all right it’s sort of wasting time isn’t it somebody else could have that appointment but if you’re actually doing that yourself you know I decide yes that I need to see someone then you know I can do that.” (R20).

Four patients however, felt that a wait of up to ten days to see someone was too long and that:

“… the only concern I have is that you always have to leave a message and wait for them to ring back and I’m one of these people that once I’ve rung I want something doing immediately” (R23).

One patient expressed disappointment at the outcome of his telephone discussion with a rheumatology nurse who did not immediately offer an appointment with a consultant which had been expected.

“She said if you are still like this in a week’s time come back and you will see XXXX. But I mean in a week’s time you know the flare had gone down so what was the point of that?” (R1).

In this instance, although the patient was dissatisfied with the initial response from the helpline, his later comment that his flare had subsided within the week suggests that the actions of the rheumatology nurse when triaging his telephone call was reasonable.

### Safety

Patient initiated systems of care have the potential for harm as well as benefit. The eligibility criteria for being suitable for DA follow-up were broad (adult, RA for at least 2 years, able to initiate phone contact) and both staff and patients raised concerns that it may not be suitable for everyone.

“Perhaps having a more consensual agreement as to who goes in and who doesn’t across the board would be helpful… you could say well all of these patients are stable-ish and have been coming for years so let’s put them all in here and see what happens…” (HCP3).

“..I don’t say it’s good for everyone because some people do get very confused about you know different things.” (R19)

Concerns were raised that some patients had not contacted the helpline and when attending the safety net review their health had obviously deteriorated. One member of staff suggested that age and social factors appeared to affect ability to utilise the service effectively:

“…the younger ones [patients] seem to drive it [DA] well… but I think for a group of patients we need to say ‘not yet’. I think we need to wait until we’ve really got past all the social issues acting on their wellbeing and thinking. I don’t think this should just be driven by disease” (HCP5).

As well as concerns that some might patients not have sufficient capability to participate, there is a related concern that others don’t see themselves as ‘candidates’ and would not call for help even in the face of significant symptoms.

“…I’ve had a bit of a flare up but its fading away so I am just sort of hanging on but [Lead Nurse] did say that after eighteen months they would call me anyway if I wasn’t in before that so I’m hanging on really” (R5).

### The everyday challenges of managing change

The idea for the service development needed organisational ‘buy in’ before it could be implemented and it had been necessary to stress the potential costs savings and waiting list management with managers rather than improvements in patient-centred care.

“…one of the set high selling points for me to the wider business management team was about the resource saving… but the idea that I was going to be seeing less people because the way that the hospital access funds is by a tariff per visit… so getting people’s mind set away from… you’ve got to get revenue” (HCP1).

Implementing the redesign was led by a core team of clinicians and didn’t involve everyone, with information being fed to colleagues outside the main group on a piecemeal basis. One member of the team explained she was not part of the core group and the way the changes were initially presented to the wider team (clinicians and administrative staff) had not been ideal.

“…there were some meetings before it [DA] was set up…I wasn’t involved in all of them…so I had sort of an bit of an idea as to what it was all about” (HCP7).

This also caused operational difficulties as the needs of the DA system were not always made clear to new staff, including those booking appointments.

“… this [problems with clinic reviews] is the problem when the booking clerk changes because the girl before knew DA very well and the next one doesn’t… there needs to be someone down in the department who solely runs DA appointments… I think this [DA] needs to be a closed shop” (HCP5).

There did not appear to be a shared decision making process regarding the suitability of patients for DA and this resulted in a lack of consensus and confusion, such as one patient group that take biological therapies receiving a mix of regular appointments based on NICE guidelines as well as using the DA system.

The need to plan for the change in workloads to different members of the team needs to be considered as DA impacted on staff workloads to run education sessions, respond to contact via the telephone helpline, arrange urgent appointments and ensure medical notes are in the right place at the right time. In addition, as patients were now attending DA clinics in response to a disease flare clinicians spoke of their struggles to complete a full review within the allocated clinic time.

“…if it [the appointment] is booked at short notice then there’s an element of racing around to get the notes and make sure the nurse has seen them…and then get them to xx [off site]” (HCP 7).

“I’m getting more direct access patients and they come up with more active complex problems perhaps more than one problem. I am realising that a 15 minute routine follow-up slot perhaps isn’t long enough to address all the issues” (HCP4).

There is also a need to have systems in place to balance continuity of care, staff absences and, where required, the need for an appointment within ten days. In order to address these issues a number of changes were made to how this was managed. The consultants had agreed that when a colleague was absent and one of their patients requested a review, the patient would be told their consultant was away and there might be limited availability with another HCP, but no promise to meet the ten day timescale was given. The patient was told they would be contacted again if a clinic time was available. This deferment gave consultants the opportunity to read their medical notes and if the case was complex they would ask for the patient to be booked in to see their own consultant when he or she returned. However, if a case was considered urgent consultants did their best to accommodate them in their DA clinics. This slight change in service was also reflected in later education sessions.

“… as the education goes on I will say ‘all your consultants take a standard 2 week holiday and therefore, if, unfortunately you call during this time you may find that your appointment is just 1 or 2 days outside the 10 day timescale’” (HCP5).

## Discussion

Putting into place new procedures within a busy, already stretched system is often challenging. In this project issues arose due to its origins from within the workforce rather than management, and due to it being perceived as different, through being a ‘research’ project. It is possible that as a result of these factors, implementing DA did not have normal management and administrative back up to iron out the inevitable snags. In order to successfully manage change there are general issues about team work, communication and ownership of change processes that need to be considered from the outset. The apparent lack of engaging the whole team early on in the process appears to conflict with the suggestion that implementation processes are framed through active participation and the collective purposive action aimed at some goal [12]. It can be difficult for clinicians to effectively bring about organisational change and management engagement and support is essential [13], particularly around the expertise needed to facilitate change.

Although Whear et al. [14] suggested that the benefits of information provision face-to-face are unclear, our study found that these were important factors. In particular, trust in the patient-nurse relationship built on familiarity with the nurse and this facilitated acceptance of the new system. Analysis suggested that the biographical and social context through which individuals experience living with a long-term, chronic medical condition and their ongoing relationships with healthcare professionals are crucial to understanding the impact of self-management education [14].

For both patients and HCPs the biggest impact of the implementation of DA clinics has been the wholesale shift in review initiation. The DA clinic therefore appears to support recent NHS guidelines that foster greater patient involvement in managing long-term illness [15] as well as NICE guidance for managing rheumatoid arthritis in adults [16] by providing rapid access to treatment and management of disease through active help seeking. Whilst it is important to note that all patients who transferred over to the new system completed an education session which informed them how DA reviews would work, none were offered a structured programme for self-management of disease as suggested in a previous trial [3] or given a guidebook to aid self-management [14].

All respondents commented favourably on the shift towards self-management and greater empowerment in the control of their disease. However, individual circumstances and needs of patients should be considered when contemplating a move to patient-driven review. It is unlikely that 100% of patients are suitable for patient-initiated care. For a DA review system to be implemented successfully, patients need to be offered management based on ability and choice rather than medical condition – those who can self-manage and are suitable for DA and others who need to stay with the traditional clinician-led outpatient care. For example, some patients do not have the confidence to ring the helpline, to recognise deterioration in their health or may feel they do not want to be a burden [17]. It may be inappropriate to offer a DA review to those with reduced cognitive capacity and those offered the opportunity but unwilling to participate can continue with traditional outpatient care [18]. Yet, paradoxically this shift in control appears to sit counter to the historical paternalistic ethos of healthcare where healthcare professionals were in control and deemed to be the ‘expert’. Now patients had been given greater autonomy over their disease management, despite the difficulties this could pose to patients who perhaps preferred control to remain with healthcare profession, or who lacked the necessary factor such as intellect, memory or communication skills to describe symptoms over the telephone [19]. However, a potential drawback of the change to a DA system is that most appointments are reactive and deal with a ‘crisis’ and the more slow and progressive aspects of a disease which require a proactive approach may be missed by both patients and clinicians.

DA can also be seen as fiscal strategy that seeks to reduce costs whilst empowering patients and attempting to improve satisfaction. However, because those seen have more active problems a DA follow-up service may mean that clinicians see fewer patients but for longer appointment times. The ‘Payment by Results’ (PbR) system used by the NHS appears to have an unintended consequence of increasing the frequency of clinical appointments [14]. It is therefore not clear how the DA system would fit into current commissioning strategies such as PbR.whether they increased or simply changed.

The study raises a question about generalisability of the findings in that it was confined to one NHS teaching hospital in the South West England - a region with the highest proportion of older people in the UK and where non-white ethnic groups make up around 5% of the region’s population [20]. All those who were transferred to DA had access to a telephone and were able to communicate effectively with a telephone answering machine in the first instance. These factors may impact on service redesign in different areas of the UK. For example in areas with greater cultural diversity multi-lingual education sessions may be required. A further limitation of this study was the lack of resources to undertake a second interview with all staff to establish how their experiences may have changed over time [21]. Serial interviews of patient participants may also have been valuable to help inform how patients’ needs may change over the course of a year of engaging with a new service model. A further limitation was not using the observation of the education session as a formal method of data collection to be analysed in conjunction with the interviews. This triangulation of data may have added a differing perspective to the findings.

## Conclusions

There are a range of factors affecting the implementation of a DA review system in rheumatoid arthritis. How service change processes are communicated to both patients and staff appears crucial to both early acceptance and sustainability of practice. Patients need to be confident that any change in service delivery will improve service outcomes and are not necessarily simply cost-saving exercises or a mechanism for removal from consultant lists. Confidence can be bolstered by the use of established and already trusted HCPs to run education sessions prior to transfer from clinician-driven to patient-initiated review systems.

Whilst patient-initiated systems of care can be of enormous benefit in allowing access to a clinician when required, they also have the potential for harm. The most obvious harm risk in terms of clinical outcomes can result from situations where a patient fails to request an appointment if their illness deteriorates [22], or the significance of a slowly progressive negative change in disease or functional state being missed by the patient. In order to ensure that patient safety is not compromised in these ways, DA clinics should include a ‘safety-net’ appointment if a patient has not been seen for a specified period of time. Clearly sustainable management of any long-term illness involves ongoing interaction between the patient and healthcare services in order to make ‘illness work’ – a shared activity beneficial to both patients and professionals [17].

We have discussed the key findings to emerge from our study and have endeavoured to offer practical solutions at both a generalisable and local level in order to overcome normative and structural constraints on the implementation processes. Based on our experience we believe that DA clinics constitute a clear service improvement for rheumatoid arthritis outpatient care and there is evidence of benefit with other long-term conditions such as inflammatory bowel disease and breast cancer [14]. Future research should be undertaken to establish both the effectiveness and cost-effectiveness of DA with a broader range of conditions in addition to establishing how best to implement DA into practice.
